# Differences in Physical Activity, Sedentary Behavior, Health-Related Physical Performance Indices and Academic Achievement: A Comparative Study of Normal-Weight and Obese Children in Qatar

**DOI:** 10.3390/jcm13041057

**Published:** 2024-02-13

**Authors:** Souhail Hermassi, Sascha Ketelhut, Ferman Konukman, Mohammed Ali Ayari, Senaid Al-Marri, Nasser Al Rawahi, El Ghali Bouhafs, Claudio R. Nigg, René Schwesig

**Affiliations:** 1 Physical Education Department, College of Education, Qatar University, Doha 2713, Qatar; fkonukman@qu.edu.qa (F.K.); sealmarri@qu.edu.qa (S.A.-M.); nalrawahi@qu.edu.qa (N.A.R.); 2 Institute of Sport Science, University of Bern, 3012 Bern, Switzerland; sascha.ketelhut@unibe.ch (S.K.);; 3 Department of Mathematics, Statistics, and Physics, College of Arts and Sciences, Qatar University, Doha 2713, Qatar; moh.ali@qu.edu.qa; 4 Department of Sports Science, Martin-Luther-University Halle-Wittenberg, 06120 Halle (Saale), Germany; bouhafs.elghali@gmail.com; 5 Department of Orthopedic and Trauma Surgery, Martin-Luther-University Halle-Wittenberg, 06120 Halle (Saale), Germany; rene.schwesig@uk-halle.de

**Keywords:** physical activity, anthropometrics, academic achievement, sedentary behavior, schoolchildren

## Abstract

**Background:** The relationship between physical activity (PA), health-related physical performance (PP), and academic achievement (AA) plays an important role in childhood. This study examined the differences in PA, sedentary behavior, health-related PP, maturity status, and AA between normal-weight and obese school children in Qatar. **Methods:** Eighty schoolchildren were recruited (age: 12.1 ± 0.6 years). Based on age-specific BMI percentiles, the children were classified as normal weight (n = 40) or obese (n = 40). Moore’s equations were used to estimate their maturity status (PHV). The measurements encompassed anthropometric data as well as PP tests (medicine ball throw, postural stability, handgrip strength). AA was assessed by reviewing school records for grade point average in Mathematics, Science, and Arabic courses. The total amount of time spent participating in PA each week was calculated using the International Physical Activity Questionnaire Short Form. **Results:** Handgrip strength was the only parameter that showed a relevant group difference (*p* < 0.001, η_p_^2^ = 0.15; normal weight: 19.7 ± 3.46 N; obese: 21.7 ± 2.80 N). We found only one moderate correlation between PHV and handgrip strength (r = 0.59). **Conclusions:** The findings suggest that obesity status alone might not serve as a sufficient predictor of AA in school or PA levels.

## 1. Introduction

Sedentary behavior and physical inactivity among adolescents and children represent a significant and growing public health concern [1,2]. An increasing number of young people are not meeting the recommended levels of physical activity (PA) [3], which can have detrimental effects on both their physical and mental health. Already in childhood and adolescence, physical inactivity is associated with a higher prevalence of obesity [4], which in turn is linked to various metabolic disorders [5].

Furthermore, there is a pervasive belief within the literature that a positive correlation exists between academic achievement (AA) and PA levels. This idea finds support in research demonstrating a beneficial relationship between PA during school hours and AA [6,7]. Moreover, higher levels of physical fitness have been associated with superior AA [6,7]. However, it is important to note that prior studies in this domain have yielded conflicting outcomes [8,9,10].

The inconsistent results can be attributed to the fact that ‘physical fitness’ is potentially an oversimplification, given that ‘fitness’ encompasses numerous physiological systems and performance metrics. Although aerobic capacity and muscular performance (e.g., agility, muscle strength, sprinting performance, flexibility) constitute the most crucial components of physical performance, it is important to recognize exercise—including low and high intensities—exerts great impacts on neurobiomarkers, brain health, and, subsequently, AA [11]. To achieve a more detailed and nuanced comprehension of how physical fitness influences AA, it becomes imperative to distinguish which facets of fitness are most closely linked to specific processes or domains.

In recent years, there has been a notable surge in research focusing on the correlation between AA and body weight, particularly body mass index (BMI) [12,13,14]. BMI finds widespread application in research and public health domains for categorizing individuals’ weight statuses due to its simplicity in assessment, scalability, and non-labor-intensive nature. Consequently, it is routinely employed in research on the relationship between AA and obesity [14].

However, the literature reveals inconsistent results [13,14,15,16]. While certain studies posit a positive correlation between AA and anthropometric parameters [13,14], others propose an inverse correlation [15,16,17], suggesting that a higher BMI might be associated with inferior AA in children. In spite of these observations, further research on the factors contributing to these conflicting results is highly warranted.

Moreover, there is limited literature addressing the underlying difference between health-related physical performance (PP) parameters, body composition, and the AA of middle school students in the Middle East [7,17,18]. It is important to elucidate these relationships in specific demographics, such as Qatari children and adolescents, because cultural differences can influence PP and AA relationships [14,17,18]. According to the recent literature, approximately 75% of schoolchildren in Qatar do not meet the daily recommended PA levels [19]. Moreover, about 55% of children spend prolonged periods on sedentary activities such as watching TV and playing video games [19]. In addition, 39% of children in Qatar are classified as overweight, with 23% falling into the category of obese or morbidly obese [19], which highlights the need for further research in this geographical region.

Considering the strong evidence supporting improved cognitive function resulting from health-related fitness [20,21,22], it is important to acknowledge the significance of adequate levels of PP for children’s AA. This holds importance as AA serves as a predictor of lifelong health and quality of life across diverse cultures [23].

Therefore, this cross-sectional study aimed to investigate the differences in PA, sedentary behavior, health-related PP, AA, and maturity status among male and female normal-weight and obese school children in Qatar. Furthermore, it examined the relationship between PP, BMI, and AA. The study hypothesized that differences in PP and AA would be apparent between normal-weight and obese school children.

## 2. Materials and Methods

### 2.1. Study Design

Convenience sampling was employed in this study, selecting participants from a single urban school in the Doha Community (Qatar), adhering to predetermined inclusion and exclusion criteria.

#### 2.1.1. Subjects

A total of 80 healthy schoolchildren (age: 12.1 ± 0.6 years; body mass: 56.5 ± 12.7 kg; height: 1.57 ± 0.09 m; body mass index (BMI): 22.7 ± 3.95 kg/m^2^, seat height: 122 ± 9 cm; arm span: 158 ± 11 cm) were included in the analysis. Children were recruited from a single school within a Doha Community (Qatar) using convenience sampling. The school involved in the study adhered to the active school policies outlined by the Ministry of Education and Higher Education in Doha. As per this policy, students were expected to have two 50 min physical education lessons each week, along with engaging in PA both in the classroom and during recess.

#### 2.1.2. Ethics

This cross-sectional study adhered to the Declaration of Helsinki and was approved by the institutional review board of Qatar University (QU-IRB 1542-FBA/21—Date of approval: 20 May 2021 and renewed on 18 June 2022) and the Ministry of Education and Higher Education of Qatar (REF: 18/2021). The study plan and objectives were communicated to both the school’s management team and physical education teachers. Prior to enrollment, students and their parents or legal guardians were provided with information about the study’s objectives and procedures and gave written informed consent.

#### 2.1.3. Inclusion and Exclusion Criteria

Children were eligible to participate if they (1) provided written informed parental or guardian consent, (2) were in good health, had no contraindications for physical activity, (3) had no physical limitations to exercise, and (4) were between 10 and 13 years old. Children were excluded if they (1) were diagnosed with a psychological disorder (e.g., depression, attention-deficit disorder, and anxiety); (2) were on medication (e.g., medication affecting the nervous system or antidepressants), (3) failed to present a signed informed consent form by their legal guardians, or (4) did not take part in regular physical education lessons.

### 2.2. Research Protocol

After obtaining consent from the parents/guardians of the children, two qualified researchers conducted and recorded field tests with the support of the school’s physical education teacher between 1 October 2021 and December 2021. Data collection took place between 08:00 AM and 10:00 each day during the physical education sessions in an indoor sports court under consistent environmental conditions (average temperature: ~28 °C–29 °C; relative humidity: 65%).

Throughout these test days, participants were instructed to maintain their usual diet. They were advised not to engage in vigorous PA, consume caffeinated beverages, or eat within 24 h, 4 h, and 2 h prior to the testing, respectively. On the first day, anthropometric parameters were obtained. The following day, the stork static balance test and handgrip strength test were performed. On the third day, the medicine ball throws were carried out. On the last day, the children completed a PA questionnaire. Two weeks following the first testing phase, test days 1 to 3 were repeated to determine test–retest reliability. The values obtained from the second test day were considered for analysis.

#### 2.2.1. Anthropometry

Body mass (model TBF 105; Tanita Corporation of America, Inc., Arlington Heights, IL, USA) and height (Holtain stadiometer, Crosswell, Crymych, Pembrokeshire, UK) were measured to the nearest 0.1 kg and 0.1 cm, respectively. Body mass (kg) × body height (m^2^) was used to compute BMI. Age and gender-specific cutoff values for BMI [24,25] were used to classify the students as normal weight (BMI: 18.0–20.9 kg/m^2^; n = 40, 20 female), or obese (BMI: >23.0 kg/m^2^; n = 40, 20 female).

#### 2.2.2. Predicted Maturity Offset

The following two gender-specific equations were applied [26] to predict participants’ maturity offset: Boys: Maturity offset (years) = −8.128741 + (0.0070346 (age sitting height)), Girls: Maturity offset (years) = −7.709133 + (0.0042232 (age stature)).

#### 2.2.3. Physical Fitness Tests

Before the PP tests, a general warm-up was conducted, which included 5 min of low-intensity running followed by various functional exercises (trunk rotation, trunk side-bends, trunk wood-chops, internal and external rotary movements of the shoulder, push-ups, and 8 to 10 ball throws) along with a 3 min passive recovery.

Postural control was assessed using the stork Balance Test [27]. The medicine ball overhead throw [28] was conducted using a 3 kg medicine ball with a diameter of 21.5 cm. A standard adjustable digital handgrip dynamometer (T.K.K. 5401, Tokyo, Japan) was used to determine the handgrip strength of the dominant hand, with a sensitivity of 10 N. Detailed descriptions of all the PP tests employed can be found in the prior literature [17].

#### 2.2.4. Academic Achievement

The students’ Grade Point Average (GPA) and subject-specific percentage scores in Arabic language, Mathematics, and Science from the first semester of the academic year 2021–2022 were used to assess their AA. In each subject, students were graded through three assessments, including an oral exam, a written examination, and a final exam. We included only Science and Mathematics because of our specific interest in science-related subjects. It has been reported that PP is particularly beneficial for subjects that rely more heavily on executive cognition, such as mathematics and science-related subjects [29]. Regarding the choice of “Arabic language”, researchers have found a strong relationship between language and learning, with a positive impact on various academic subjects [30].

#### 2.2.5. Physical Activity and Sedentary Behavior

PA was evaluated using the IPAQ—SF (International Physical Activity Questionnaire—Short Form). Details regarding the administration and scoring techniques of the IPAQ—SF can be found elsewhere [31]. The reliability and validity of IPAQ—SF have been established in diverse populations across different countries [2,31]. The results were used to quantify the daily amount of PA, expressed in terms of the Metabolic Equivalent of Task minutes per day (MET min/day). PA was divided into three intensities based on the IPAQ—SF scoring protocol [31]: moderate (4 METs), vigorous (8 METs), and walking (3.3 METs) [30,31]. The total PA was calculated as the sum of the three intensities. Additionally, the time spent sitting was calculated to quantify sedentary behaviors [31]. A recent study has used the IPAQ—SF to estimate the PA levels in children and elementary middle schools aged from 9 to 13 years [29,32].

### 2.3. Statistical Analysis

All variables were examined for normal distribution (Shapiro–Wilk Test) and variance homogeneity (Levene Test for Equal Variances) before conducting statistical analysis. All variables are presented as mean ± standard deviation (SD), minimum and maximum values, and 95% confidence intervals (95% CI). Mean differences in anthropometric and performance parameters between groups (normal weight vs. obese and female vs. male) were tested using a two-factor (sex and weight) univariate general linear model [33]. Differences between means were considered statistically significant if *p*-values were <0.05 and partial eta-squared (η_p_^2^) values were >0.15 [34].

A sample size calculation (nQuery Advisor 4.0; Statistical Solutions, Saugus, MA, USA) was performed using previous data [35]. Considering the primary outcome parameter—which was Science—and employing a *t*-test for independent groups, a mean difference of 7.4 (pooled SD: 8.00; d = 0.89), a significance level set at 5%, and a power of 80% it was determined that a sample size of 21 participants in each group would be required [33]. Pearson’s product–moment correlations were calculated to determine the relationship between parameters of different dimensions (anthropometric, PP, AA, PA). The magnitude of correlation (r) between the variables were: classified using the following criteria: <0.1, trivial; 0.1–0.3, small; 0.3–0.5, moderate; 0.5–0.7, large; 0.7–0.9, very large; and 0.9–1.0, almost perfect.

Statistical analysis was performed using SPSS version 28.0 for Windows (SPSS Inc., IBM, Armonk, NY, USA).

## 3. Results

### 3.1. Normal Distribution and Variance Homogeneity

The variables arm span (*p* = 0.082) and handgrip strength (*p* = 0.155) displayed a normal distribution. In terms of variance homogeneity, eight parameters (body mass: *p* < 0.001; BMI: *p* = 0.005; postural stability: *p* = 0.007, handgrip strength: *p* = 0.003, vigorous minutes: *p* = 0.016, vigorous MET: *p* = 0.013, walking MET: *p* = 0.041, total MET: *p* = 0.047) did not show homogeneity of variance.

### 3.2. Variance Analysis

#### 3.2.1. Anthropometry

The anthropometric parameters, weight (*p* < 0.001, η_p_^2^ = 0.60), and BMI (*p* < 0.001, η_p_^2^ = 0.80), showed significant differences between the groups (Table 1). The parameters seated height (*p* = 0.925), height (*p* = 0.226), biological maturation (*p* = 0.432), and arm span (*p* = 0.778) did not demonstrate any significant difference between the groups (normal weight vs. obese). At a descriptive level, apart from arm span (normal weight/female) and BMI (obese/female), the male subjects exhibited higher values across all anthropometric parameters.

#### 3.2.2. Health-Related Fitness and Academic Achievement

A significant and relevant difference (*p* < 0.001, η_p_^2^ = 0.15, d = 0.64) was observed between the two groups for hand grip strength (Table 2). The obese group (21.7 ± 2.80 N) attained higher values than the normal-weight group (19.7 ± 3.46 N). On a descriptive level, obese boys consistently showed the highest performance level in all PP parameters. For AA, we calculated the highest values for normal-weight boys (Table 2).

#### 3.2.3. Physical Activity

**Figure 1:**
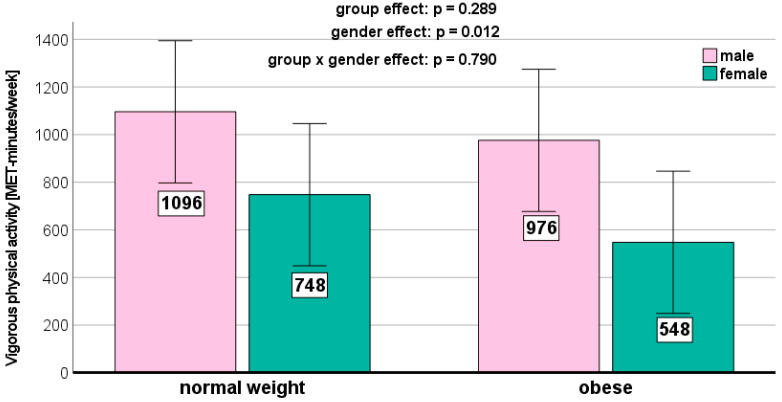
Vigorous physical activity depending on BMI and gender.

**Figure 2:**
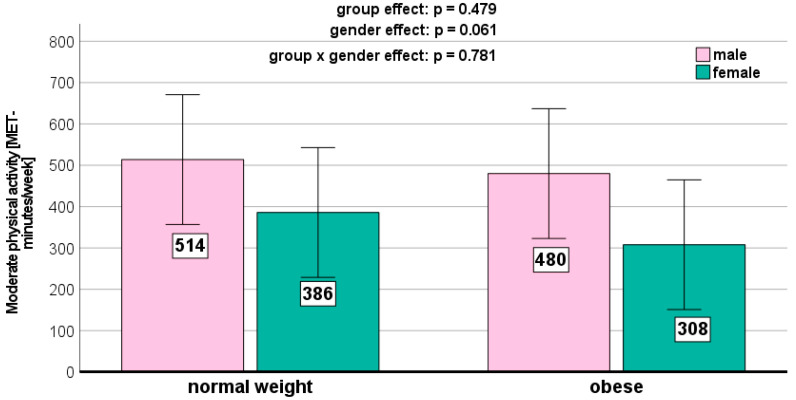
Moderate physical activity depending on BMI and gender.

**Figure 3:**
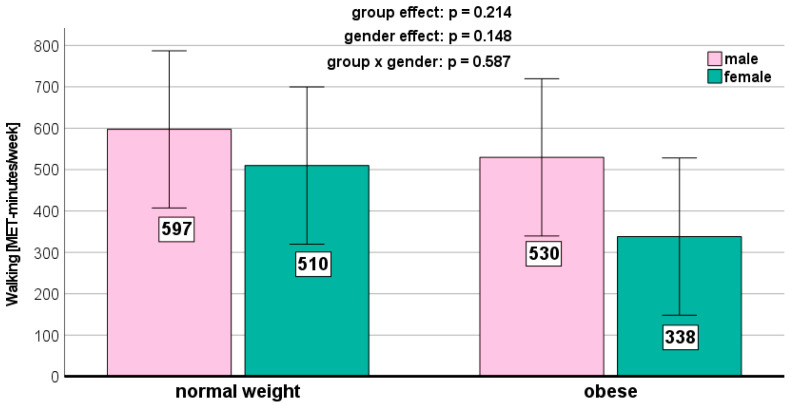
Walking time per week depending on BMI and gender.

**Figure 4:**
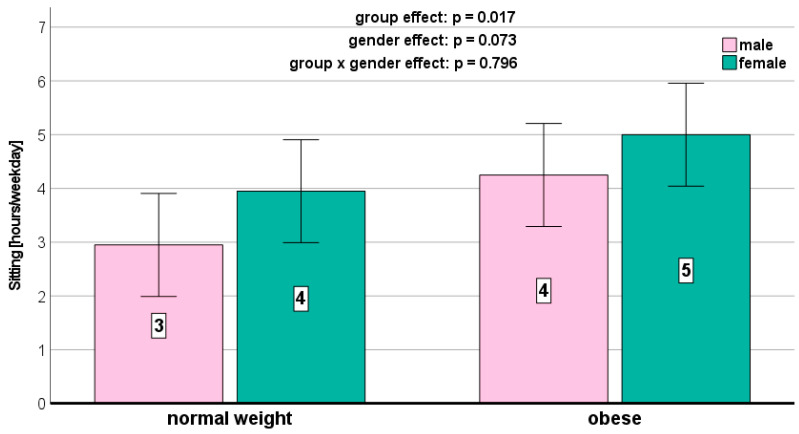
Sitting time per weekday depending on BMI and gender.

**Figure 5:**
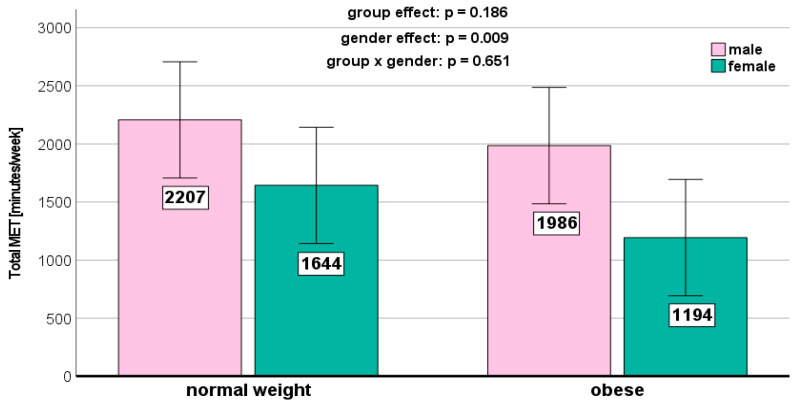
Total MET per week depending on BMI and gender.

We could not detect any significant weight-related effects on the PA parameters (Figure 1, Figure 2, Figure 3, Figure 4 and Figure 5). Gender effects were determined for vigorous PA (*p* = 0.012, Figure 1) and the total MET (*p* = 0.009, Figure 5). On a descriptive level, boys generally exhibited higher levels in all PA parameters. Girls in both groups spent slightly more time sitting (Figure 4).

### 3.3. Correlations between Parameters of Several Dimensions

**Figure 6:**
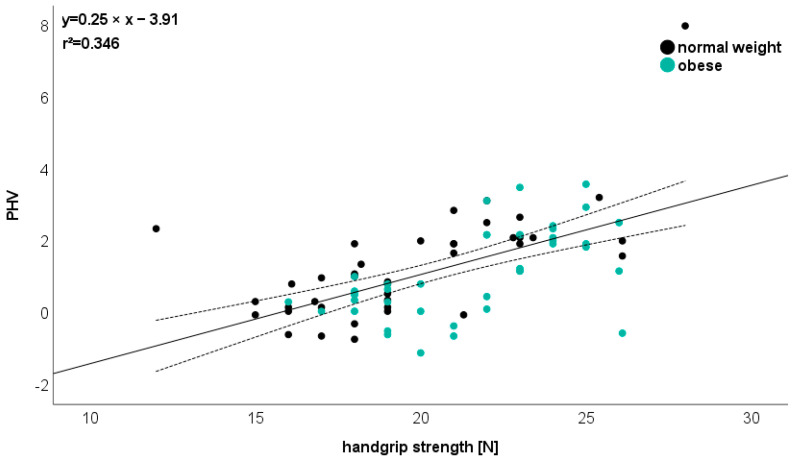
Relationship between biological maturation (PHV) and handgrip strength. Please note that one dot can represent several subjects.

Only one significant (r > 0.5) product–moment correlation (Pearson) was identified between biological maturation (PHV) and handgrip strength (r = 0.588, Figure 6).

## 4. Discussion

The aim of this study was to examine the differences in PA, sedentary behavior, health-related PP, and AA between normal-weight and obese middle school students in Qatar. Notably, one of the PP (handgrip strength) but none of the AA and PA parameters exhibited significant differences between the groups. However, in terms of descriptive values regarding AA, male subjects of the normal-weight group showed the highest values. For PP, it was the males from the obese group who displayed the highest performance level. Between the different dimensions, only one relevant relationship was identified (PHV vs. handgrip strength).

### 4.1. Physical Activity and Sedentary Behavior

PA is essential for individual well-being and health. Engaging in regular PA provides numerous benefits, including decreased levels of anxiety and depression, along with a lowered risk of developing non-communicable diseases [35].

We did not detect any significant weight status-related effects on the PA parameters in the present study. This aligns with previous research [36,37,38]. However, other studies reported conflicting results [37,38]. Descriptively, the normal-weight children in our study exhibited the highest levels of moderate (467 ± 376 MET-minutes/week) and vigorous (964 ± 745 MET-minutes/week) PA. Normal-weight children also reported the highest MET-minutes per week for walking (627 ± 503 MET-minutes/week) and demonstrated the longest sitting time (4.55 ± 2.41 h per week).

According to the present results, there were no significant gender differences in sedentary behavior. This conflicts with previous research that has reported that boys exhibit lower levels of sedentary behavior compared to girls [39,40].

Similarly, there were no significant differences between boys and girls regarding PA levels in the present study. This, again, conflicts with previous research in Europe [41,42], the United States [43], and Australia [44]. The disparity between our study and other research may be attributed to cultural differences among students within Middle Eastern society. However, upon examining regional data, a gender disparity in PA engagement becomes apparent. Studies from Gulf Cooperation Council countries [45], show that the proportion of schoolchildren meeting 60 min MVPA/day is lower among girls (4 to 39%) than boys (44 to 71%). In a recent study conducted by Hermassi et al. [29] among 9-year-old schoolchildren in Qatar, it was found that boys exhibited higher levels of PA in comparison to girls. The conflicting results could be attributed to a combination of social, methodological, and socioeconomic factors. Nevertheless, additional research is necessary to validate these assumptions.

### 4.2. Health-Related Fitness

In school physical education lessons, fundamental skills such as short sprints, direction changes, balance, and throwing are acquired and applied [46]. Previous research has indicated a negative relationship between being overweight or obese and PP [14,47,48,49]. However, in the present study, female students with normal weight exhibited the lowest performance levels across all PP parameters except for the stork balance test. On the other hand, the male obese group demonstrated the highest performance levels in the medicine ball throw and stork balance test.

The higher performance in medicine ball throw in the obese group may be explained by the fact that the BMI is influenced by body height and mass, which play an important role in body proportions and are key determinants of muscle strength [50,51,52,53]. In this regard, the study by Esmaeilzadeh and Kalantari [54] adjusted for body fat mass and found that being overweight or obese was related to enhanced performance in several PP tests, such as the standing long jump, run speed tests, and sit and reach test. This suggests that the level of fat mass itself may not be the sole determinant of PP.

In child development, the ability to maintain postural control plays a crucial role, serving as a fundamental prerequisite to performing complex motor skills and a variety of physical movements [54,55]. In the present study, the normal-weight group exhibited a higher postural performance than the obese group. This observation is noteworthy because postural control has been recognized as a significant intrinsic factor that heightens the risk of falling and sustaining injuries among youth and children [56,57].

Muscular strength is often measured via handgrip strength. In the current sample, the obese group (21.7 ± 2.80 N) achieved higher values for handgrip strength compared to the normal-weight group (19.7 ± 3.46 N). These values are higher than the results reported by Palacio-Agüero et al. [58] or by García-Hermoso et al. [59]. The difference in hand grip strength between the obese and the normal-weight groups in the present study may be attributed to variations in body composition and maturation stage [60,61].

### 4.3. Academic Achivement

There is substantial evidence suggesting a positive impact of physical health on mental and cognitive functions that are linked to AA [62,63]. Moreover, AA has been shown to predict future health and quality of life across various cultures [64]. This has led policymakers and researchers to acknowledge the importance of maintaining adequate levels of physical health in schoolchildren to support AA.

In the current study, normal-weight boys showed the highest values of AA, while obese girls displayed the lowest AA level (Arabic and Science). These findings are consistent with a recent study conducted by Hermassi et al. [17,56], which reported similar group differences in AA, specifically in Mathematics and Science, between obese and non-obese 13-year-old schoolchildren. However, the observed group difference for the Arabic language in the present study did not reach statistical significance The current findings align with earlier studies that suggested enhanced AA among students with normal weight and have higher PP levels [20,23,47]. However, other studies report a positive correlation between AA and anthropometric parameters [13,14], and no association between obesity and AA in school-aged children [62,63].

While the reasons for the inconsistent results remain unclear, differences in the methods used to measure obesity (criteria: BMI or body fat), growth stages, analysis techniques, or participant characteristics [65,66] may offer potential explanations for some of the uncertainty. Furthermore, if previous studies have interpreted results solely on the alpha level and used an arbitrary threshold of *p* < 0.05 with small sample sizes, it is possible these studies fell afoul of a type II error.

Variations in results could also stem from the utilization of diverse strategies for assessing AA. This could help explain discrepancies, given that obesity has not been linked to lower test scores [67], yet it has been associated with lower GPA [68]. This is problematic because one might think that lower test scores would translate into a lower GPA, but it does highlight (a) the necessity of specifying the metrics used to measure academic performance and (b) the multifactorial nature of the relationship between obesity and GPA because it is not just a matter of lower test scores.

### 4.4. Biological Maturation

Biological maturation represents the progression inherent in human growth and development that affects all tissues, organs, and systems of the body [69]. Previous research indicates an association between obesity and maturation in both boys and girls, showing a positive correlation in girls and a negative correlation in boys [70]. However, our study yielded intriguing results as we found no significant differences in biological maturation, as determined by PHV, between normal-weight and obese boys and girls. Additionally, we observed no discernible relationship between PHV and PA. These findings are contradictory to previous research suggesting that children’s participation in PA may be influenced by their maturity status, as it affects both psychosocial and biological elements [71].

Regarding AA and health-related PP, no significant correlation could be detected in the present study. This conflicts with the study of Hermassi et al. [14] Showing that the aerobic fitness, muscular strength, and change-of-direction ability had a positive, linear relationship with academic scores. In addition, Xu et al. [72] reported that muscle strength and aerobic endurance of primary school students were correlated with their academic performance.

In our study, only for handgrip strength could a correlation with PHV be detected across all participants. This is in line with Yapici et al. [73] for children aged 12 years. The main finding of this study was that biological maturation was associated with right- and left-hand grip strength. However, the inconsistent results could potentially be explained by variations in age, geographical location, and cultural differences.

### 4.5. Practical Implications

In summary, this study represents the first investigation into the complexities surrounding PA, sedentary behavior, health-related PP, AA, and maturity status among both normal-weight and obese children in Qatar. Thus, this research offers valuable insights for public health initiatives and future studies. Moreover, in addressing the inconsistencies observed in previous research, this study aims to elucidate conflicting outcomes by acknowledging the oversimplification of PP. Additionally, this research contributes crucial data to a geographic region with limited available information. These outcomes will support multi-sectoral efforts, including collaborations in physical education among ministries of health, sports, youth, and education.

### 4.6. Limitations

It is essential to acknowledge certain limitations that may influence the interpretation of the study results. First, this study did not account for several potential confounders, including factors such as the school environment, socioeconomic status, or children’s ethnicity. Second, due to the study’s cross-sectional design, causality cannot be established. Third, to differentiate between fat mass and lean mass, body fat should be measured. Fourth, we did not follow the commonly accepted standards for being overweight or obese. Rather, the sample was segregated into groups based on BMI stratification.

## 5. Conclusions

This study investigated the differences in PA, PP, and AA among BMI-stratified normal-weight and obese schoolchildren. Surprisingly, the children’s weight status did not significantly affect their PP, AA, or PA levels. Descriptively, it was observed that obese boys displayed higher levels of PP compared to their normal-weight peers, while, conversely, normal-weight boys reported the highest AA.

Future studies might consider employing the gold standard for assessing body composition, while also incorporating additional variables such as biological maturation status to further validate our observations and enhance accuracy. Despite the lack of a definitive connection between weight status, PP, and AA, parents, schools, and physical education teachers in Qatar should collaboratively explore strategies to enhance engagement in PA and improve health-related PP to address a pressing public health concern.

## Figures and Tables

**Table 1:** Comparison of anthropometric parameters between normal-weight and obese children. Values are given as mean ± SD. Significant and relevant effects (effect criteria: *p* < 0.05 and η_p_^2^ > 0.15). Performance maxima marked in bold.

Parameters	Normal Weight (n = 40)			Obese (n = 40)			Variance Analysis	
	Male (n = 20)	Female (n = 20)	Total (n = 40)	Male (n = 20)	Female (n = 20)	Total (n = 40)	*p*	η_p_^2^
**Height (m)**	1.57 ± 0.07	1.55 ± 0.07	1.56 ± 0.07	**1.61 ± 0.09**	1.56 ± 0.11	1.59 ± 0.10	0.226	0.02
**Weight (kg)**	48.9 ± 5.51	44.9 ± 3.16	46.9 ± 4.86	**68.1 ± 9.43**	64.2 ± 11.4	66.1 ± 10.5	**<0.001**	**0.60**
**Body mass index (kg/m²)**	19.8 ± 1.14	18.7 ± 1.33	19.2 ± 1.34	26.1 ± 2.09	**26.3 ± 2.34**	26.2 ± 2.19	**<0.001**	**0.80**
**Seat height (cm)**	**122 ± 17.7**	121 ± 3.57	122 ± 12.8	122 ± 2.75	121 ± 4.92	122 ± 3.96	0.925	0.00
**Arm span (cm)**	156 ± 10.9	**159 ± 11.2**	157 ± 11.0	159 ± 10.5	157 ± 10.4	158 ± 10.4	0.778	0.00
**Biological maturation** **PHV (year)**	**2.33 ± 1.52**	0.29 ± 0.62	1.31 ± 1.54	2.18 ± 0.71	0.11 ± 0.58	1.14 ± 1.23	0.432	0.01

**Table 2:** Comparison of health-related fitness and academic achievement parameters depending on body between normal-weight and obese children. Values are given as mean ± SD. Significant and relevant effects (effect criteria: *p* < 0.05 and η_p_^2^ > 0.15). Performance maxima marked in bold.

Parameters	Normal Weight (n = 40)			Obese (n = 40)			Variance Analysis	
	Male (n = 20)	Female (n = 20)	Total (n = 40)	Male (n = 20)	Female (n = 20)	Total (n = 40)	*p*	η_p_^2^
**Physical performance parameters**								
**Medicine ball throw (m)**	3.55 ± 0.58	2.83 ± 0.51	3.19 ± 0.65	**4.03 ± 0.87**	3.26 ± 0.58	3.64 ± 0.83	0.002	0.12
**Stork balance test (s)**	2.03 ± 0.30	1.75 ± 0.61	1.89 ± 0.49	**2.06 ± 0.60**	1.81 ± 0.52	1.93 ± 0.57	0.718	0.01
**Handgrip test (N)**	21.5 ± 3.86	17.8 ± 1.51	19.7 ± 3.46	**23.8 ± 1.24**	19.5 ± 2.20	21.7 ± 2.80	**<0.001**	**0.15**
**Academic achievement parameters**								
**Arabic**	**89.8 ± 8.50**	83.3 ± 6.34	86.5 ± 8.10	81.3 ± 7.59	84.0 ± 8.21	82.6 ± 7.93	0.027	0.06
**Mathematics**	**89.8 ± 9.10**	87.0 ± 8.65	88.4 ± 8.87	85.0 ± 8.43	83.5 ± 8.29	84.3 ± 8.29	0.036	0.06
**Science**	**90.8 ± 9.36**	85.0 ± 10.0	87.9 ± 9.99	84.0 ± 9.26	83.0 ± 7.85	83.5 ± 8.49	0.036	0.06

## Data Availability

The raw data supporting the conclusions of this article will be made available by the authors without undue reservation.
